# Genome-wide evaluation of genetic diversity and linkage disequilibrium in winter and spring triticale (x *Triticosecale* Wittmack)

**DOI:** 10.1186/1471-2164-13-235

**Published:** 2012-06-12

**Authors:** Katharina V Alheit, Hans Peter Maurer, Jochen C Reif, Matthew R Tucker, Volker Hahn, Elmar A Weissmann, Tobias Würschum

**Affiliations:** 1 State Plant Breeding Institute, University of Hohenheim, Stuttgart 70593, Germany; 2 Centre of Excellence in Plant Cell Walls, University of Adelaide, Urrbrae, SA 5064, Australia; 3 Saatzucht Dr. Hege GbR, Domäne Hohebuch, Waldenburg 74638, Germany

## Abstract

**Background:**

Recent advances in genotyping with high-density markers nowadays enable genome-wide genomic analyses in crops. A detailed characterisation of the population structure and linkage disequilibrium (LD) is essential for the application of genomic approaches and consequently for knowledge-based breeding. In this study we used the triticale-specific DArT array to analyze population structure, genetic diversity, and LD in a worldwide set of 161 winter and spring triticale lines.

**Results:**

The principal coordinate analysis revealed that the first principal coordinate divides the triticale population into two clusters according to their growth habit. The density distributions of the first ten principal coordinates revealed that several show a distribution indicative of population structure. In addition, we observed relatedness within growth habits which was higher among the spring types than among the winter types. The genome-wide analysis of polymorphic information content (PIC) showed that the PIC is variable among and along chromosomes and that especially the R genome of spring types possesses a reduced genetic diversity. We also found that several chromosomes showed regions of high genetic distance between the two growth habits, indicative of divergent selection. Regarding linkage disequilibrium, the A and B genomes showed a similar LD of 0.24 for closely linked markers and a decay within approximately 12 cM. LD in the R genome was lower with 0.19 and decayed within a shorter map distance of approximately 5 cM. The extent of LD was generally higher for the spring types compared to the winter types. In addition, we observed strong variability of LD along the chromosomes.

**Conclusions:**

Our results confirm winter and spring growth habit are the major contributors to population structure in triticale, and a family structure exists in both growth types. The specific patterns of genetic diversity observed within these types, such as the low diversity on some rye chromosomes of spring habits, provide a basis for targeted broadening of the available breeding germplasm. In addition, the genome-wide analysis of the extent and the pattern of LD will assist scientists and breeders alike in the implementation and the interpretation of association mapping in triticale.

## Background

Modern genomic approaches such as association mapping rely on a detailed characterisation of the population structure and linkage disequilibrium (LD) present in the species under study. LD is the non-random association of alleles at different loci and, similar to genetic diversity, is affected by several genetic factors such as recombination, mutation and genetic drift [1]. Other factors that influence LD include selection for favourable alleles [2,3], domestication [4,5], outcrossing of crop cultivars with genetically distinct lines of wild ancestors and landraces [6], and admixture [7]. LD between markers and QTL is the basis for association mapping analyses and the distance over which LD stretches defines the number of markers required to cover the genome [8]. The range over which LD stretches and its rate of decay may vary significantly between chromosomal regions due to different breeding history. The effect of LD range on association mapping, however, is double-edged: long-ranging LD reduces the number of markers required to cover the entire genome but also entails a low mapping resolution [9].

For this reason, it is important to assess and understand the extent and patterns of LD in the species under investigation. In the area of small grain cereals, studies on LD are available for wheat [10,11], rye [12] and barley [13,14], but to date are lacking for triticale. A detailed understanding of population structure and relatedness is of crucial importance for breeding as well as for association mapping as it is considered to be one of the major reasons for spurious marker-phenotype associations [15,16]. Recent advances in genotyping with high-density markers enable genome-wide genomic analyses. Diversity Arrays Technology (DArT) markers [17] have previously been identified as a valuable tool in cereals and have been employed successfully to create linkage maps of triticale [18] as well as its parents wheat [19-22] and rye [23,24].

Triticale (x *Triticosecale* Wittmack; 2n = 6x = 42), a partially outcrossing small grain cereal, was created by mankind through hybridisation of wheat and rye. It is a crop with broad genetic potential and is widely adapted to abiotic stress conditions such as aluminium toxicity, drought, salinity, acidic or waterlogged soils [25,26]. Due to its valuable composition of amino acids and stable performance in less productive environments, triticale has furthermore attained importance as quality feed stock [27,28]. Triticale produces more biomass for a comparable grain yield than other crops and, therefore, can increase the industrially useable biomass for bioenergy and biofuels without increasing competition with food production on arable land [29].

In this study we used the previously reported triticale-specific DArT array combining markers developed in wheat, rye and triticale [30] to analyze population structure as well as the extent and the pattern of LD in a worldwide set of triticale lines. In particular the objectives of this study were to (i) investigate the population structure and genome-wide genetic diversity in a set of 161 winter and spring triticale lines, (ii) determine the extent and pattern of LD, and (iii) to draw conclusions on the prospects of genome-wide association studies (GWAS) in triticale.

## Results

### Population structure and kinship

**Figure 1:**
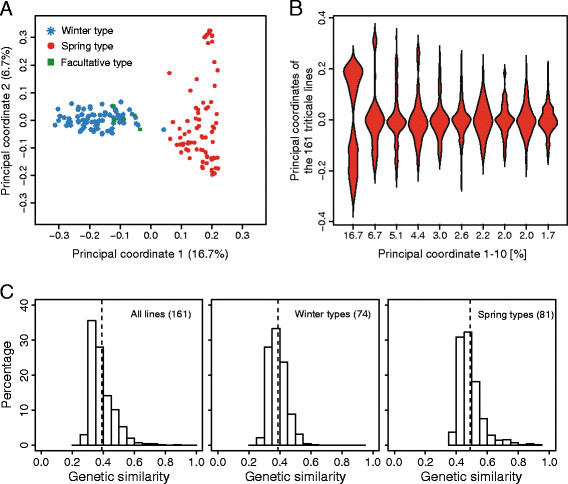
**Analysis of population structure and familial relatedness.** ( **A**) Principal coordinate analysis of the 161 genotypes based on modified Rogers’ distances. Percentages in parentheses refer to the proportion of variance explained by the principal coordinate. ( **B**) Violin plot showing the density distribution of the first ten principal coordinates. Percentages on the x-axis refer to the proportion of variance explained by the respective principal coordinate. ( **C**) Histogram of the genetic similarities (simple matching coefficient) among all lines, winter types and spring types. Dashed lines indicate the population-specific means.

Our study was based on a set of 161 diverse triticale lines of worldwide origin including those used in a recent study by Badea *et al.*[30]. Genome-wide DArT marker data were used to assess the population structure by a principal coordinate analysis (PCoA) based on the modified Rogers’ distances of the individuals. The first two principal coordinates explained 16.7% and 6.7% of the total genetic variation (Figure 1A). A violin plot of the density distributions of the first ten principal coordinates revealed that several show a distribution indicative of population structure, especially the first principal coordinate (Figure 1B). The first principal coordinate divides the triticale population into two clusters according to their growth habit. The second principal coordinate further differentiates mainly spring types. The subgroups, winter and spring types, were were clearly separated whereas the few facultative types included in this study clustered with the winter group. The cultivar Matinal which is classified as winter type was close to the spring types. The assessment of the genetic similarity among winter and spring types revealed a higher degree of relatedness among the spring (0.49) than among the winter types (0.39) (Figure 1C).

### Patterns of genetic diversity

**Figure 2:**
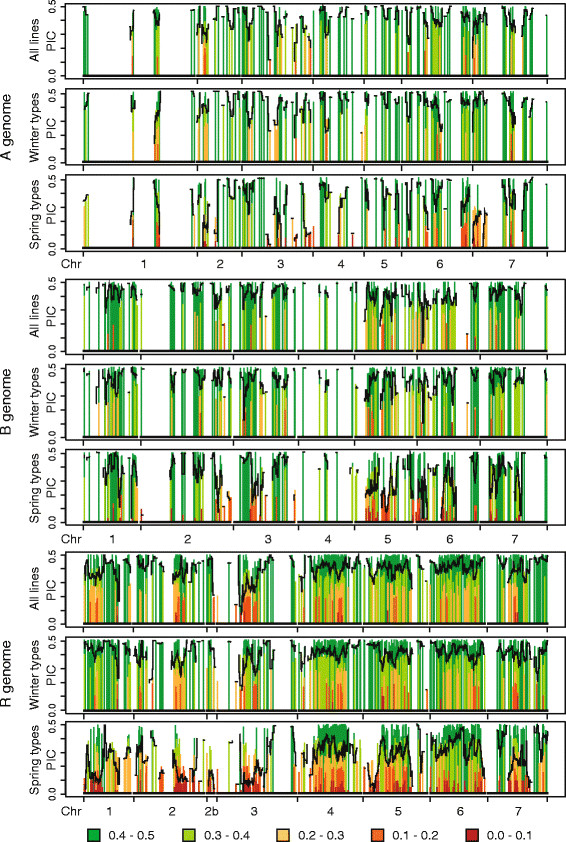
**Polymorphic information content (PIC) along the chromosomes.** PIC values along the chromosomes separately for the A, B and R genome among all lines (161), winter types (74), and spring types (81). Different colours indicate varying levels of the PIC and black lines indicate the mean PIC assessed by a sliding window approach.

Genetic diversity was assessed by calculating the polymorphic information content (PIC) for all markers (n = 2,079) (Table 1). PIC values varied slightly among genomes and considerably among and along chromosomes (Figure 2). The mean PIC of the entire set of lines was 0.40 across all three genomes and 0.40, 0.42 and 0.38 for the A, B and R genome, respectively. Chromosome 4B showed the highest average PIC in spring as well as in winter types (0.45 and 0.43), whereas 3R showed the lowest (0.35 and 0.14). For facultative types the lowest average PIC was observed on chromosome 1A and the highest on 1R. Averaged across all chromosomes, spring and facultative types (0.30 and 0.34) showed a lower PIC than winter types (0.40).

**Table 1:** Polymorphic information content (PIC) per chromosome

**Chr**^**a**^	**1**			**2**				**3**			**4**			**5**			**6**			**7**			
	**A**	**B**	**R**	**A**	**B**	**R-1**	**R-2**	**A**	**B**	**R**	**A**	**B**	**R**	**A**	**B**	**R**	**A**	**B**	**R**	**A**	**B**	**R**	**Mean**
All lines (161)	0.40	0.44	0.37	0.36	0.44	0.39	0.40	0.38	0.43	0.29	0.43	0.46	0.40	0.45	0.38	0.40	0.41	0.38	0.41	0.40	0.43	0.40	**0.40**
Winter (74)	0.37	0.42	0.43	0.40	0.42	0.37	0.40	0.39	0.40	0.35	0.41	0.45	0.40	0.43	0.39	0.41	0.41	0.41	0.40	0.42	0.42	0.40	**0.40**
Spring (81)	0.32	0.39	0.16	0.26	0.34	0.19	0.17	0.29	0.38	0.14	0.39	0.43	0.33	0.38	0.24	0.28	0.34	0.30	0.35	0.28	0.34	0.28	**0.30**
Facultative (6)	0.23	0.32	0.44	0.31	0.33	0.33	0.31	0.32	0.36	0.35	0.25	0.42	0.31	0.35	0.36	0.30	0.37	0.31	0.31	0.40	0.39	0.36	**0.34**

^a^ Chr = Chromosome.

Numbers in brackets refer to the number of genotypes in the respective group.

### Genetic differentiation between winter and spring triticale

**Figure 3:**
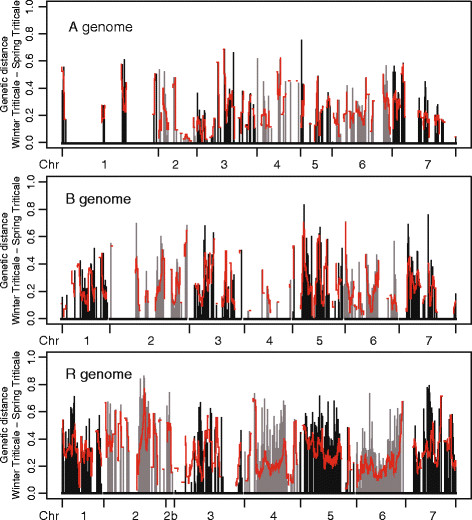
**Genetic distance between winter and spring types.** Genetic distance between winter and spring types assessed for every marker. Grey and black colours separate different chromosomes (Chr), red lines indicate the mean genetic distance assessed by a sliding window approach (5 cM window used at 500 positions along the chromosomes).

In order to identify chromosomal regions harbouring QTL for traits that are under divergent selection between the two growth habits, the genetic distances between winter and spring types were calculated for each locus and plotted along the chromosomes. As shown in Figure 3, there were several chromosomes with regions of high genetic distance between the two growth habits. Some of the regions exhibited a pattern with smaller genetic distances at the flanks which increased towards higher peaks in the centre. Peaks were observed on chromosomes 1A, B, R, 2R, 3R, 4R, 5B, R, 6A, B, R, 7A, B, R. In general, the highest genetic differences which extended over large regions were detected on chromosomes from the R genome.

### LD across the genome and along chromosomes

The extent and distribution of linkage disequilibrium (LD) was estimated for the entire set of triticale lines as well as for the winter and spring types in order to identify differences in the triticale genome among the two growth habits. For the entire set of lines the population-specific threshold for LD due to linkage (95% quantile of *r*^2^ values of 2,008,200 pairs of unlinked loci) equaled 0.12, for the winter types 0.11, and for the spring types 0.17.

**Figure 4:**
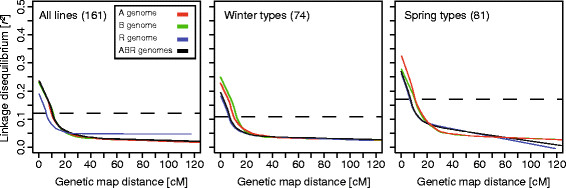
**Decay of linkage disequilibrium (LD) with genetic map distance.** Curves were fitted by locally weighted regression smoothing and show that LD decays with genetic map distance for the A, B, R and the ABR genome among the 161 genotypes, winter types (74) and spring types (81). Dashed lines indicate the population-specific thresholds for LD due to linkage.

The association of LD and genetic map distance indicated that intrachromosomal LD decayed with a different rate depending on the growth habit and on the genome (Figure 4). To evaluate the extent of LD we used the intersection of the fitted regression curve with the respective population-specific threshold for LD due to linkage. Regarding all lines, the A and B genomes showed a similar LD of 0.24 for closely linked markers and a decay below the threshold within approximately 12 cM. The LD in the R genome was lower for closely linked markers with 0.19 and decayed much faster within a distance of approximately 5 cM. This difference in LD extent and decay between the A and B genomes on the one side and the R genome on the other was also observed within the winter and spring types. A noticeable difference between the two growth habits, however, was the extent of LD for closely linked loci which was higher for spring types than for winter types. Consistent with this, we observed a higher average LD between adjacent markers for the spring types (0.45) compared to the winter types (0.39). Despite this higher extent of LD, the range was comparable between winter and spring types.

**Figure 5:**
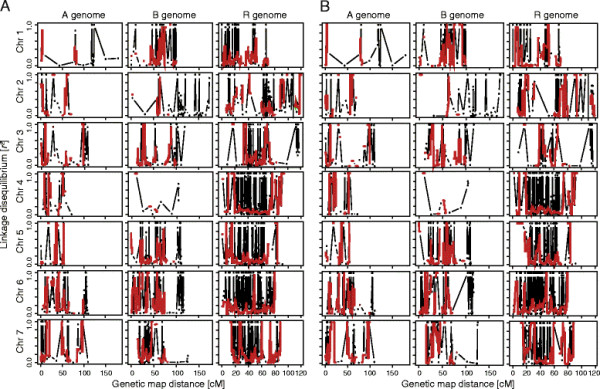
**Distribution of linkage disequilibrium (LD) along the chromosomes.** Distribution of LD along the chromosomes in ( **A**) winter types and ( **B**) spring types. Black points show the LD of each locus with its neighbouring locus. Red line represents the median of *r*² values calculated by a sliding window approach (5 cM window used at 500 positions along the chromosomes).

Further to this, we examined the distribution of LD along the chromosomes in winter and spring triticale by a sliding window approach (Figure 5). Large differences in LD were detected between chromosomes, but with comparable patterns between winter and spring types. On several chromosomes (*e.g.*, 1R, 5B, 5R) the pattern was comparable but the LD higher in spring types. In addition, we detected three genomic regions (2R, 6R, 7B) with noticeable differences in LD between the two growth habits.

## Discussion

Knowledge about the genetic diversity, population structure and the extent and pattern of linkage disequilibrium is important for crop improvement strategies, as well as for the design and the analysis of association mapping studies [1,15,16,31]. In addition, such information is indicative of the population history shaped by breeding and selection. In our study, we present data for a large set of 161 diverse winter and spring triticale genotypes assayed with 2,079 genetically mapped, genome-wide distributed DArT markers.

### Population structure and genetic diversity in triticale

The DArT markers used in this study have been assessed as an efficient tool to investigate genetic diversity in triticale [30] and in addition have been mapped to an integrated linkage map [18]. We found that the first principal coordinate, which explains 16.7% of the genetic variance, clearly separates the winter and the spring types (Figure 1A). Whereas the winter types were mainly separated with regard to the first principal coordinate, the variation among spring types was explained by the second principal coordinate. This separation of winter and spring types has been reported for triticale [30] as well as for other crops including wheat [10], barley [13] and rapeseed [32]. An exception was the French winter type Matinal which, with regard to the first principal coordinate, was closer to the spring types. This is in accordance with results of Badea *et al.*[30] and can be explained by the origin of this cultivar which included a cross to the spring cultivar Colossal. In our study, the facultative genotypes clustered among winter types which was already reported for the French cultivar Bienvenu by Badea *et al.*[30] and could be explained by their pedigree and the requirement for winter hardiness. The presence of two sub-populations in hexaploid triticale, corresponding to the different growth habits, is not surprising and reflects the different breeding goals and programmes. Compared to the separation of gene pools in maize, which explains almost 50% of the genetic variance [33], both winter and spring types in triticale are relatively closely related due to a short and partly common breeding history and the existing gene flow between both growth habits.

We observed a higher genetic similarity among spring types (Figure 1C) which is in contrast to the study of Chao *et al.*[10] in wheat. CIMMYT is considered as a major developer and supplier of improved spring triticale germplasm for agricultural research around the world and many advanced lines trace back to the early line Armadillo and a cross between Armadillo and Maya 2 [34,35] potentially explaining the lower genetic diversity. Genetic diversity in crops was confined by the domestication bottleneck and has since been enhanced by mutation and crosses among divergent lines. It must be noted here that in contrast to other crops, the bottleneck for genetic diversity in the man-made crop triticale is the genetic diversity of the durum wheat and rye lines used to establish primary triticale. The higher genetic diversity among winter types observed in this study may thus reflect a broader panel of parents used to establish the winter types as compared to the spring types or a higher number of backcrosses with bread wheat.

### Patterns of gene diversity

Polymorphic information content (PIC) is a measure of allele frequencies at single loci or summed or over multiple loci. For biallelic markers such as DArTs, the PIC ranges from 0 to 0.5, where 0.5 indicates equal allele frequencies and 0 fixation of one allele. Our estimates of the PIC regarding single chromosomes varied from 0.14 to 0.46 with an overall mean of 0.40 (Table 1), which is higher compared to that of wheat with a mean PIC of 0.2 [10]. Our results are in accordance with those of Badea *et al.*[30] who used genotypes and markers that partly overlap with our study and who reported a mean PIC for triticale of 0.37. Furthermore, our results on the mean PIC of the A (0.40), B (0.42) and R (0.38) genomes are also in agreement with those of Badea *et al.*[30]. These results are in contrast to those of Tams *et al.*[27] who reported higher PIC levels for triticale in general and higher levels for the A and B genomes compared to the R genome. Kuleung *et al.*[25] also reported higher PIC estimates of the wheat (0.55) and rye (0.53) genomes. The differences in PIC values are likely attributable to the different types of markers used in the studies as Tams *et al.*[27] used multiallelic SRR markers as compared to the biallelic DArTs used by us and Badea *et al.*[30]. In contrast to the results of Chao *et al.*[10] in wheat, who observed a higher PIC for the winter types as compared to the spring types, we found the mean PIC of the winter types exceeded that of the spring types for all chromosomes. This result is in accordance with the genetic similarity estimates (Figure 1C) and might be explained by the establishment and the breeding history of spring triticale.

We further exploited the information contained in the PIC values and investigated their distribution along the chromosomes (Figure 2). This analysis revealed that especially for the R genome, the PIC values are not constant along chromosomes but show strong variation. We observed chromosomal regions with clusters of low PIC values (*e.g.*, chromosomes 7A, and 1R-5R in spring types), mainly on chromosomes of the R genome and within spring types. Chromosomes exhibiting reduced PIC values harbour genomic regions with limited polymorphism possibly due to selection for QTL located in these regions, or due to the reduced diversity for these regions among founder lines. Our results suggest that only few, or genetically similar rye lines, have been used for the establishment of primary spring type triticale. The consequence of this reduced degree of polymorphism observed for the R genome of spring types is that little variation will be created with crosses among lines and that only a fraction of the genetic variation offered by the rye genome is exploited in spring triticale. Possible solutions are crosses with winter types or the creation of new primary spring triticale with more diverse rye lines.

### QTL underlying growth habit differentiation

The principal coordinate analysis revealed that the winter and spring growth habit contributes the major source of population structure in triticale (Figure 1A). We therefore reasoned that differences in allele frequencies between the two growth habits could be employed to map QTL under differential selection between winter and spring types. Several regions showed strong differences in allele frequencies (Figure 3). The regions on homologous groups 2, 5 and 7 might be associated with growth habit traits such as vernalization (vrn) requirement, flowering time, or cold tolerance. From studies of wheat it is known that genes on homologous group 2 are involved in photoperiod sensitivity, earliness *per se* (eps) and vernalization requirement, genes on group 5 in heading date, eps and vernalization, and chromosomes 7A and 7B are predicted to contain one *Vrn* gene each [36]. Furthermore in rye, the spring growth habit gene *Sp1*[37] and members of the *Cbf* ( *C-repeat Binding Factor*) gene family, which are likely to be involved in cold regulation and possibly interconnected with vernalization [12], were reported to be located on chromosome 5R and QTL for flowering time on 2R and 7R [38]. Surprisingly, we only detected a small peak on chromosome 5A where the major gene for flowering time regulation, *Vrn1*, is known to be located [39]. This might be due to an insufficient coverage of the genetic map to detect differences on chromosome 5A [18]. Another possible reason is that different *Vrn1* alleles might have been fixed for a long time, possibly even in the founder lines used for the establishment of primary triticale, such that recombination events have had sufficient time to re-shuffle the genome surrounding *Vrn1* and consequently no more long-ranging differences are detectable. Furthermore, it is also possible that the observed differences in allele frequencies can arise due to different selection in the breeding programmes ( *e.g.*, resistances or quality traits) or randomly by genetic drift.

### Extent of linkage disequilibrium in triticale

Linkage disequilibrium is affected by different genetic factors [1] and is the basis for association mapping approaches, which mainly detect indirect associations between markers and the trait. The extent and the pattern of LD are therefore of high interest for genomics research in triticale. We observed a higher population-specific threshold for LD due to linkage in the spring types (0.17) than in the winter types (0.11) which is in accordance with results from wheat [10,11] and rye [12]. The thresholds observed in wheat, however, were lower than those observed in this study, which may also be attributed to the different marker types or variation in sample size. The higher rate of LD between unlinked loci observed for triticale may be due to an enhanced selection for epistatic QTL or to genetic relatedness due to predominant parents in the germplasm. The latter is likely to be the case in the subgroup of spring types where some parents (*e.g.*, Erizo_9, Pronghorn) were used in several crosses [30]. In triticale, genetic relatedness can also arise if certain founder lines were predominantly used for the establishment of primary triticale.

In agreement with previous studies in wheat, rye and other crops [8,10,12,40-42] our results show a decay of LD with genetic map distance. The LD decay was variable among growth habits and genomes as indicated by the distance at which the fitted LD curves intersect the population-specific threshold (Figure 4). Irrespective of the genome, the initial LD value was lower and the decay slightly faster in the winter types compared to the spring types. In addition, we observed a faster decay of LD on the R genome (5–8 cM) than on the A and B genomes (~12 cM) which may be attributed to historical LD caused by the different mating systems of the parents of triticale; rye (outcrossing) and wheat (selfing). Hao *et al.*[43] also reported LD decay within distances of 5–10 cM in modern bread wheat varieties whereas Chao *et al.*[10] found higher initial LD values for both growth habits. A direct comparison of LD in triticale with results in rye was not possible due to a lack of published studies or the fact that they were restricted to the candidate gene level and used different types of markers [12]. Compared to fully outcrossing crops such as maize [8,44] triticale shows a relatively slow decay of LD as the man-made creation of the crop constituted a severe bottleneck and generated LD that has not been broken down during its short breeding history.

### Patterns of linkage disequilibrium

The marker density underlying this study is sufficient to warrant a high-density genome-wide analysis of LD. Analyzing the extent and patterns of LD in germplasm of interest provides additional information for the design and the interpretation of association mapping studies [8]. Our results revealed that LD patterns are variable along chromosomes in winter as well as in spring types (Figure 5). Variable LD along chromosomes was also found for other crop species such as maize, wheat, and sugar beet [8-12]. Regions containing LD blocks might harbour QTL responsible for agronomically important traits and therefore a reduced decay of LD in this chromosomal region. Chao *et al.*[10] attribute the divergence in the extent of LD between hexaploid wheat populations to unique breeding histories and selection pressures applied to genes located in the different genomes during the process of cultivar development. This might in part also explain the results obtained in our study. Interestingly, we also observed three genomic regions on chromosomes 2R, 6R and 7B with divergent LD patterns between winter and spring types pointing to differences in the recent history of the two populations.

### Prospects for genome-wide association mapping in triticale

In human genetics an LD threshold of r² ≥ 0.8 is desired to capture nearly all common variation in the genome in GWAS [45]. For triticale, the highest currently available marker density is provided by DArT markers which have been mapped to an integrated linkage map [18,30]. Based on these markers we observed LD between closely linked markers of approximately 0.2-0.3 and between adjacent markers of approximately 0.4. This indicates that association mapping is feasible in triticale with the restriction that mainly large effect QTL can be detected given the available marker density. In order to detect QTL with medium or small effect size the marker density must be increased further for example by genotyping-by-sequencing approaches [46].

In conclusion, our results suggest that LD in triticale shows a medium decay with genetic map distance, thus limiting the achievable mapping resolution. It must be noted, that as a consequence of the observed variable LD along chromosomes the mapping resolution in association mapping studies will be variable. Our results, however, also imply a certain degree of LD caused by relatedness, population stratification or genetic drift, which can cause false marker-phenotype associations and therefore must be accounted for in association mapping studies.

## Conclusions

Our results, based on a set of 161 worldwide winter and spring triticale lines genotyped at high-density with DArT markers, confirm winter and spring growth habit as major source for population structure in the triticale germplasm. Furthermore, our results suggest a family structure in the sub-populations, which both should be taken into account when performing genome-wide association mapping studies.

The genome-wide analysis of LD revealed that LD is variable among genomes but also along individual chromosomes. This must be taken into consideration as it strongly influences the mapping resolution in GWAS. The DArT marker assay available for triticale can be considered as sufficient for the detection of large effect QTL. As some chromosomal regions show only low marker coverage or a fast decay of LD with genetic map distance, novel approaches such as genotyping-by-sequencing may be required for the detection of small effect QTL.

## Methods

### Plant material and molecular markers

This study was based on a diverse worldwide set of 161 triticale (x *Triticosecale* Wittmack) lines. A subset of these (133) have been described in Badea *et al.*[30]. Of the 161 lines, 74 are winter types, 81 are spring types, and 6 are facultative types (Additional file 1). The lines were genotyped with the current triticale DArT marker array [30] by Triticarte Pty Ltd, Yarralumla ACT, Australia [47] (Additional file 1). The DArT markers used have recently been mapped in a triticale integrated consensus map [18]. The genome representation was as follows: 306 DArT markers on the A genome, 502 on the B genome, and 1,271 on the R genome. For information on the distribution of these DArT markers among and along chromosomes see Additional file 2.

### Population structure and linkage disequilibrium analyses

Associations among the 161 genotypes were analyzed by applying principal coordinate analysis (PCoA) [48] based on the modified Roger’s distances (MRD) of the individuals [49]. Polymorphic information content (PIC) was applied to assess genetic diversity and was calculated for single loci as PIC = 1 - (*p*^2^ + *q*^2^), where *p* and *q* denote the frequencies of the two alleles.

Linkage disequilibrium (LD) was assessed pairwise by the squared allele–frequency correlations *r*^2^[50] and statistical significance of LD was tested with Fisher’s exact tests [51]. The association between LD decay and genetic map distance was summarised by fitting a curve by locally weighted regression smoothing [52] to *r*² values that were plotted against the genetic map distance according to the triticale consensus map [18]. To obtain a threshold of *r*² above which LD was likely to be caused by genetic linkage the 95% quantile derived from the distribution of the *r*² values of unlinked loci was taken as a population-specific critical value [11]. The intersection of the fitted curve of *r*² values with this threshold was considered as the estimate of the range of LD in the respective genome. For the sliding window approaches a 5 cM window was used at 500 positions along the chromosomes. LD and PCoA computations were performed with the software package Plabsoft [53].

## Competing interests

The authors declare that they have no competing interests.

## Authors' contributions

KVA performed parts of the statistical analyses and drafted the manuscript. JCR participated in the design of the study and edited the manuscript. HPM, MRT, VH, and EAW edited the manuscript. TW participated in the design of the study, performed parts of the statistical analyses and helped to draft the manuscript. All authors read and approved the final manuscript.

## Supplementary Material

Additional file 1 **List of genotypes and genotypic data.** Excel spreadsheet containing the list of genotypes used and their genotypic DArT marker data. Additional data are published by Badea *et al.*[30]. Click here for file

Additional file 2 **Distribution of markers among and along chromosomes.** Excel spreadsheet containing the list of markers used for anlyses and their position on the respective chromosome. Click here for file
